# Quantitative analysis of proteomic changes in two monoclonal suspension MDCK cell lines infected with human influenza A virus (H1N1)

**DOI:** 10.1371/journal.pone.0327939

**Published:** 2025-10-21

**Authors:** Jan Küchler, Tilia Zinnecker, Patrick Hellwig, Maximilian Wolf, Dirk Benndorf, Yvonne Genzel, Udo Reichl

**Affiliations:** 1 Max Planck Institute for Dynamics of Complex Technical Systems, Bioprocess Engineering, Magdeburg, Saxony-Anhalt, Germany; 2 Otto von Guericke University Magdeburg, Bioprocess Engineering, Magdeburg, Saxony-Anhalt, Germany; 3 Universtität Bielefeld, Multi-Dimensional Omics Data Analysis, Bielefeld, North Rhine-Westphalia, Germany; 4 Applied Biosciences and Process Engineering, Anhalt University of Applied Sciences, Köthen, Saxony-Anhalt, Germany; National Taiwan Ocean University, TAIWAN

## Abstract

Suspension MDCK cells are a substrate for producing influenza A virus (IAV) and typically show very high virus yields compared to other animal cells. Due to the significant heterogeneity within cell populations, studying and comparing clonal cell lines with regard to specific properties, such as superior growth or higher productivity, could facilitate process optimization. In this study, we analyzed the expressed proteins of two clonal cell lines to identify intrinsic characteristics of effective IAV producers. We compared proteome changes in two human IAV PR8 (H1N1, A/PR/8/34) infected monoclonal suspension MDCK cell lines: C59, a low-yield IAV producer with fast cell growth and small cell diameter, and C113, a high-yield IAV producer with average cell growth and large cell diameter. We examined growth rate, size, metabolism and IAV production. A total of 5177 host cell proteins were detected in both cell lines using DIA-PASEF mode with a TimsTOFpro mass spectrometer. Analysis of the differentially expressed proteins revealed that fatty acid oxidation and branched-chain amino acid degradation were upregulated in highly productive cells. In contrast, steroid biosynthesis and DNA replication were more active in faster-growing cells. Following infection, 122 proteins were significantly upregulated (p < 0.05, log_2_-fold change ≥1) in the high-producing cell line. These proteins were associated with membrane trafficking, interactions with the IAV-NS1 protein and virus production. Additionally, 98 proteins associated with antiviral pathways such as the proto-oncogenic receptor tyrosine kinase MET and tumor necrosis factor (TNF) signaling were downregulated (p < 0.05, log_2_-fold change ≤1). In the cell line that produced lower IAV PR8 titers, 77 proteins were downregulated and 57 were upregulated after infection. RNA metabolism appeared to be downregulated, while the tricarboxylic acid (TCA) cycle and the stress response were both upregulated. In the high-yield C113 clone, only proteins associated with apoptosis and the target of rapamycin kinase (TOR) were expressed following infection. This may indicate a more effective release of virus particles. A comparison of intracellular IAV PR8 protein levels demonstrated that M1 and NA levels were 4-fold and 8-fold higher, respectively, for the high-yield C113 cell line. These findings again suggest an improved virus release.

## Introduction

Most influenza vaccines are still produced using embryonated chicken eggs. However, animal cell culture technology offers several advantages over egg-based production. These advantages include faster production times during pandemics, improved scalability, enhanced immunogenicity, better process control, and the ability to operate in closed systems [1–5].

Vero cells and Madin-Darby canine kidney cells (MDCK) are among the most promising candidates for the cell culture-based production of influenza viruses [6,7]. MDCK cells are used to produce Flucelvax Tetra (CSL Seqirus), for example, and are generally considered as a preferred substrate due to their high susceptibility and yields for a wide range of influenza viruses [3,4,8–12]. Additionally, suspension cell lines offer several advantages for industrial viral vaccine production because they can be grown to high densities, enabling large-scale production. Furthermore, chemically-defined media that are free of animal components are available [5,12,13].

From work on CHO cells, it is known that there are significant differences in cell lines due to the passaging and cultivation history. When switching to cell line development and generation of cell clones, clear differences are observed between clones and cell populations. Selecting specific cell clones for specific needs has become standard for recombinant protein production. However, this is not yet widely considered for virus production [4,5]. MDCK cell populations are also described as highly variable with respect to their growth properties, metabolism, virus titer, antigen glycosylation patterns and morphology [13–17]. Due to this inherent heterogeneity, clone selection can be leveraged for effective influenza A virus (IAV) production [6]. To analyze and compare the properties of clonal cell lines, proteomic analysis via mass spectrometry (MS) can be applied. Compared to other analytical methods, proteomics uniquely captures the cellular status, activated signaling pathways and metabolic shifts inside the cell during IAV infection. Quantitative MS, in particular, allows for an in-depth analysis of proteins produced during cell growth and IAV replication. This enables a comprehensive comparison of different cell lines [18–21]. Thus far, we have employed this approach to compare the IAV proteins produced by different cell lines cultivated in different media [22].

In this study, we use our previously described proteomic workflow for human IAV PR8 (H1N1, A/PR/8/34) protein production and expand it to cover the proteome of two IAV-infected clonal MDCK cell lines. As previously reported, clones C59 and C113 exhibit distinct characteristics regarding growth, metabolism, and virus production [6]. To improve our understanding of the high-yield clone C113, we analyzed these two different cell lines at the proteomic level. Our goal was to track IAV PR8 protein production and identify key host cell factors and metabolic pathways associated with high IAV titers in MDCK cells. Using this approach, we detected over 5000 host cell proteins in both cell lines and mapped them to several hundred metabolic pathways. This allowed us to compare the two cell lines comprehensively. Despite their origin from the same parental cell line and growth under identical cell culture conditions, our results demonstrate significant differences between the two monoclonal MDCK cell lines.

## Materials and methods

### Cells and viruses

The two MDCK clones C59 and C113, developed and provided by Sartorius (Germany) and characterized in previous studies [6], were used for all experiments. These two clones were obtained through single-cell cloning from adherent MDCK cells (#CCL-34, ATCC) and were adapted to suspension growth [6]. The monoclonal cell lines were cultivated in 4Cell® MDXK CD medium (Sartorius, Germany), supplemented with 8 mM L-glutamine. Cells were maintained in non-baffled shake flasks (Corning, #431143) with a working volume of 30 mL and cultivated at 37°C and 5% CO2, with a shaking frequency of 120 rpm (50 mm throw) in a Multitron Incubator shaker (50 mm shaking orbit, Infors AG, Switzerland). Passaging was performed 2–3 times per week, and cells were inoculated at viable cell concentrations (VCCs) ranging from 2E + 05 - 8E + 05 cells/mL. Cell viability, diameter, and concentration were measured using a Vicell XR automated cell counter (Beckman Coulter, #731050).

For infection, human influenza A virus (H1N1, A/PR/8/34, Robert-Koch institute (RKI), Berlin, Germany, 3.0 log_10_ (HA units/100 µl), 1.10E + 09 TCID_50_/mL) seed virus adapted to adherent MDCK cells (#84121903, ECACC) was used. Virus stock was diluted to achieve a multiplicity of infection (MOI) of 3. Trypsin (Thermo Fisher Scientific, no. 27250−018) was added at time of infection (final activity of 20 U/mL). A medium exchange was performed 1 h post infection (hpi) to remove remaining seed virus. Samples were taken before infection (mock) or at 12 hpi based on preliminary experiments.

### Analytics

Virus production was monitored using the hemagglutination (HA) [21] and a 50% tissue culture infectious dose (TCID_50_) assay to determine the total and infectious number of virions produced, respectively. For both assays, samples were centrifuged (3000 x g) and the virus-containing supernatant was collected and stored at −80°C before analysis. Total virus particle concentrations (C_vir_) were estimated using the erythrocyte concentration (C_ery_) in the HA assay [20]. The standard deviation of the HA assay was ± 0.081 log_10_ (HA units/100 µL). Infectious virus particle concentrations were determined using TCID_50_ assay, as described by Genzel and Reichl [21]. In this assay, adherent MDCK cells were infected with serial dilutions of the virus-containing samples in the presence of 50 units/mL trypsin. The cells were stained with an HA-specific antibody (anti-influenza A/PR/8/34 H1N1 HA serum sheep (NIBSC 03/242)) 48 hpi and virus dilutions of the sample that resulted in IAV production were identified using florescence microscopy. The dilution error of the TCID_50_ assay was 0.3 log, as reported by Genzel and Reichl [21].


$$c_{vir}=c_{ery}\cdot {\text{1}\text{0}}^{{log}_{\text{1}\text{0}}(\frac{HA}{\text{1}\text{0}\text{0}\;\mu L})}$$ (1)

### Proteomics workflow and peptide quantification

**Fig 1:**
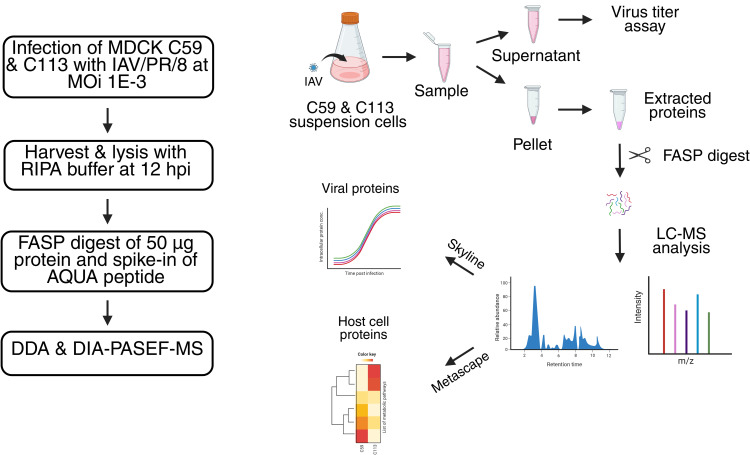
Workflow for the proteomic study comparing the C59 and C113 MDCK cell clones and IAV PR8 production. RIPA: radio-immunoprecipitation assay; FASP: filter-aided sample preparation; AQUA: absolute quantification; DDA: data-dependent acquisition; DIA: data-independent acquisition; PASEF: parallel accumulation serial fragmentation. (Adapted from [22]).

Five IAV proteins (hemagglutinin (HA), nucleoprotein (NP), neuraminidase (NA), matrix protein 1 (M1) and non-structural protein 1 (NS1)) were quantified over a single infection cycle using absolute protein quantification (AQUA) as described by Küchler et al. 2025 [22] (Fig 1). A minimum of three AQUA peptides were used for quantification of each protein. Infected cells were collected 12 hpi and processed as follows: First, the cell pellets were first washed once with 1 ml PBS. Then, they were lysed by adding RIPA buffer (Thermo Fisher Scientific, Waltham, Massachusetts, USA) and pushing them through 26G needles (Terumo Agani, Tokyo, Japan). Approximately 300 µl of lysis buffer was used for every 4E + 06 cells. Protein concentrations were determined using a commercial BCA (bicinchoninic acid) assay (Thermo Fisher Scientific, Waltham, Massachusetts, USA), and 50 µg of each sample was used for further processing and preparation for MS measurements using a modified filter-aided sample preparation (FASP) method [23].

Proteolytic digestion was performed using 2.5 µg (1:20) of MS-approved trypsin for 16 hours at 37°C. Dried samples were then diluted in 90 µL of load A buffer (Liquid chromatography-MS grade water + 0.1% trifluoric acid) plus 10 µL of AQUA standard mix (0.2 pmol/µL of each peptide). AQUA peptides with isotopic labels were used to quantify IAV peptides by back-calculating to the absolute copy number after adding a specific amount to each sample. The AQUA standard mixture contained 20 peptides for the five major IAV proteins with a heavy label (all C and N atoms of the last amino acid, either lysine or arginine, were replaced with their C13 or N15 isotopes) (Thermo Fisher Scientific, Waltham, Massachusetts, USA). For each sample, 2 µL (1 µg protein, 0.04 pmol AQUA peptides) were injected into the LC-MS/MS system (see below), enabling the quantification of approximately 1.0E + 08 protein copies/cell using the Skyline software, as determined by previous experiments and mathematical modelling approaches [22,24].

Liquid chromatography (LC) and MS measurements were performed using an UltiMate 3000 system (Thermo Fisher Scientific, Waltham, Massachusetts, USA) and a TimsTOFpro (Bruker Daltonik, Bremen, Germany). The MS was operated in data-dependent (DDA) Parallel Accumulation Serial Fragmentation (PASEF) mode and in data-independent (DIA)-PASEF mode as described before [25]. Windows for DIA-PASEF scans were determined based on DDA measurements using py_diAID (version 0.030) as described in Skowronek et al. [25] resulting in a 99.80% precursor coverage (S1 Fig). A spectrum library was created and the raw data analyzed with the fragpipe software (version 22.0) and DIA-NN software (version 2.0.2) as described before [26]. The reference genome used for the spectrum library was “*C. l. familiaris*” downloaded from Uniprot (5/9/2025). The resulting DIA-NN files (.parquet) were filtered by q-value (Q.value and Global.Q.Value) and a pivot table was created in R Studio (version 2023.06.2 build 561) using an in-house script. The protein groups were functionally annotated using Prophane [27].Subsequent comparison of relative protein expression between the cell lines was performed in Excel; significance was tested by using a Students t-test (p < 0.05, two-tailed, unequal variances). All detected proteins and their abundancies, as well as those visualized in volcano plots and heatmaps are listed in S2 File. Principal coordinate analysis (PCoA) was performed and visualized with Python (S1 File) using Numpy, (1.26.4), Scikit-learn (1.4.2), Scipy (1.13.0) and matplotlib (3.8.4). For protein network analysis, we used the online tool STRING (string-db.org; [28]) with *H. sapiens* or *C. l. familiaris*. Venn diagrams, volcano plots and heat maps were created using the respective add-ins in OriginPro (version 9.6.0.172). Protein expressed only in the infected or mock samples were analyzed using the Metascape tool (metascape.org, [29]).

This article does not contain any studies with human participants or animals performed by any of the authors.

## Results and discussion

### Growth characteristics and virus production of two different monoclonal cell lines

**Fig 2:**
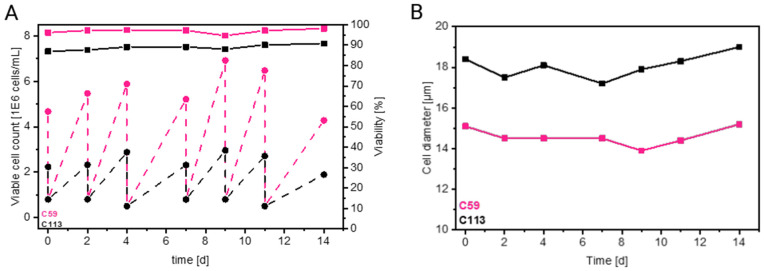
Cell growth of the C59 and C113 MDCK cell clones (C59: rose, C113: black) over several passages. A: viable cell concentration (dashed line) and viability (solid line). B: cell diameter. Both cell lines were seeded at a constant cell concentration of 5E06 - 8E + 06 cells/mL, with a seeding interval of 2 - 3 days.

First, the cell growth and virus production of the two monoclonal MDCK cell lines, C59 and C113, were compared to confirm previously observed differences [6]. The C59 clone exhibited faster cell growth, achieving a viable cell concentration that was 2–3 times higher at each passage, with high viability (Fig 2A). The diameter of the C59 cell clone was on average about 3–4 µm smaller compared to C113 (Fig 2B), which was in line with previous observations [6]. Assuming the cells were spheres, this resulted in a 1.6-fold surface area difference and a 2-fold volume difference. However, both are within the normal range for MDCK cells [15]. Note: for these calculations, we assumed a smooth surface. If there are a lot of microvilli, the real surface area could be much larger and the differences between the cell lines more pronounced. This would affect the metabolism, IAV budding and virion release [30–32].

Furthermore, the total (HA assay) and infectious (TCID_50_) virus titers were determined for both cell lines at 12 hpi (Table 1). At 12 hpi, virus production reached its plateau in MDCK cells infected at an MOI of 3. All viral processes such as protein production, replication, and release, reached their maximum rates as reported before [22,33]. The clone C113 produced 0.9 log_10_(HAU/100 µL) more virions and about 9-fold more infectious IAV, which is consistent with earlier reports [6]. Together with the higher VCCmax of the C59 clone, this results in a significantly higher cell-specific virus yield for C113. When the difference in cell size is taken into account (volume-specific virus yield), a substantially higher viral yield is also observed for C113 (Table 1). This demonstrates that, in addition to cell size, intrinsic factors may also contribute to the difference in virus yield as it was also shown in a previous study on MDCK cells [15]. Moreover, the C113 clone produces higher proportions of infectious virions than C59 (smaller ratio of CSVY_HA_/CSVY_TCID_). Overall, compared to our previous studies, the values obtained in this study are within a similar range [4,6].

**Table 1:** IAV production of the monoclonal MDCK cell lines C59 and C113. Three biological replicates from different pre-cultures were infected at a VCC of 2E + 06 cells/mL using human influenza A virus (H1N1, A/PR/8/34, RKI) at an MOI of 3, and samples were taken at 12 hpi. For significance testing, student’s t-test were performed (two-sided, unequal variances) using Excel. Cell volume was calculated from cell diameter assuming a spherical geometry. The cell-specific virus yield (CSVY_HA_) was calculated as described in [6]. Virus titers refer to maximum titers observed. VCC is defined as the maximum VCC observed during the cultivation and infection phases.

Parameter	C59	C113	p-value	Fold change
Average cell diameter [µm]	14.5	18.2	–	1.2
Cell surface [µm^2^]	656	1037	–	1.6
Average cell volume [µm^3^]	1580	3139	–	2.0
VCC_12hpi_ [*1E6 cells/mL]	2.48	2.21		
Total cell volume (VCC_12hpi_ * cell volume) [*1E6 µm^3^/mL]	3913	6927		1.8
HA titer [log_10_(HAU/100 µL)]	2.23 ± 0.17	3.13 ± 0.09	<0.01	–
CSVY_HA_ [virions/cell]	1394 ± 338	6243 ± 1917	<0.05	4.5
Volume-specific virus yield_HA_ [virions/µm^3^]	0.13 ± 0.04	0.24 ± 0.05	<0.05	1.8
TCID_50_ [*1E8 TCID_50_/mL]	1.08 ± 0.66	9.87 ± 2.71	<0.05	–
CSVY_TCID_ [virions/cell]	41 ± 24	359 ± 19	<0.05	8.8
Volume specific inf. virus yield_TCID_ [inf. virions/µm^3^]	1.88E-03 ± 1.2	1.78E-02 ± 0.49	<0.05	9.5
CSVY_HA_/ CSVY_TCID_ [total virions/inf. virions]	34	17		2

### Influenza A virus protein production of the two different monoclonal cell lines

**Fig 3:**
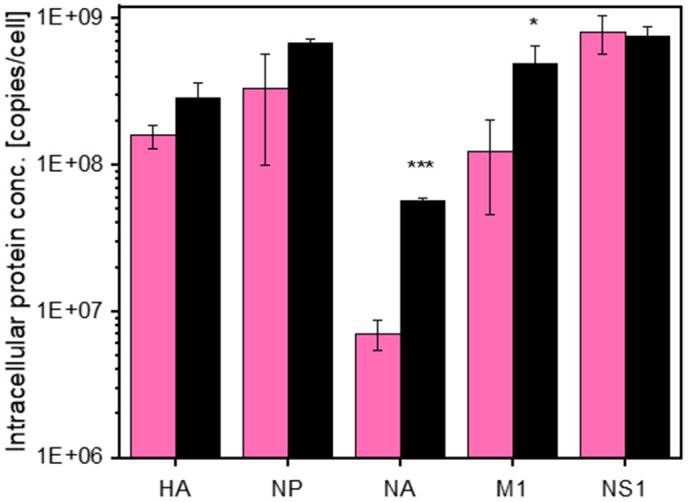
Intracellular IAV protein production of the MDCK cell clones C59 (rose) and C113 (grey). Absolute protein copy numbers for five IAV proteins at 12 hpi were determined based on isotope-labelled peptides [22]. Cells were seeded in trypsin-containing MDXK medium in shake flasks with a working volume of 30 mL, and directly infected at 2.0E + 06 cells/mL with human influenza A virus (H1N1, A/PR/8/34, RKI) using an MOI of 3. Samples were taken at 12 hpi. A complete medium exchange was performed at 1 hpi. Selected peptides were quantified by adding a defined amount of AQUA standard peptides into each sample using DIA-PASEF-MS, and analyzed with the Skyline software. A list of the labelled peptides including the limit of detection and the limit of quantification is provided in the S1–S3 Tables. Significance was tested with a two-sided student’s t-test (*p < 0.05, ***p < 0.001).

In order to compare the two monoclonal cell lines on a molecular level, the intracellular IAV protein production was analyzed. For this, the absolute protein copy numbers of the five most abundant IAV proteins during the infection process were determined by employing a previously published assay from our group [22]. As shown in Fig 3, the majority of IAV proteins were produced at comparable intracellular levels by both cell lines, which is consistent with previously published findings [22]. The concentrations of HA, NP and NS1 proteins were almost identical for both cell lines with less than 2-fold difference, mostly with slightly higher concentrations for the C113 clone. Nevertheless, a 4-fold increase in M1 protein concentrations and a 8-fold increase in NA protein concentration was observed in the C113 clone. The higher concentrations of NA protein observed in C113 could potentially favor virus assembly and budding since both proteins play a critical in the efficient release of progeny virions [34]. Furthermore, the ratio of HA to NA protein (HA/NA balance) plays a very important role in the virus production [35]. The higher copy numbers of HA compared to NA in the C59 clone could lower the rate of virus release. Furthermore, the M1 protein plays a pivotal role in virus budding. It is associated with the HA and NA proteins and it binds to the viral RNA complexes [36].

### Host cell protein analysis of the two different monoclonal cell lines

First, differences in host cell protein expression, irrespective of their relative abundances were compared between the cell lines. Therefore, DIA-PASEF measurements of the samples were analyzed with *C. l. familiaris* proteome using fragpipe and DIA-NN to create a spectral library. The latter was then processed with an R script and the online tool Prophane.

**Fig 4:**
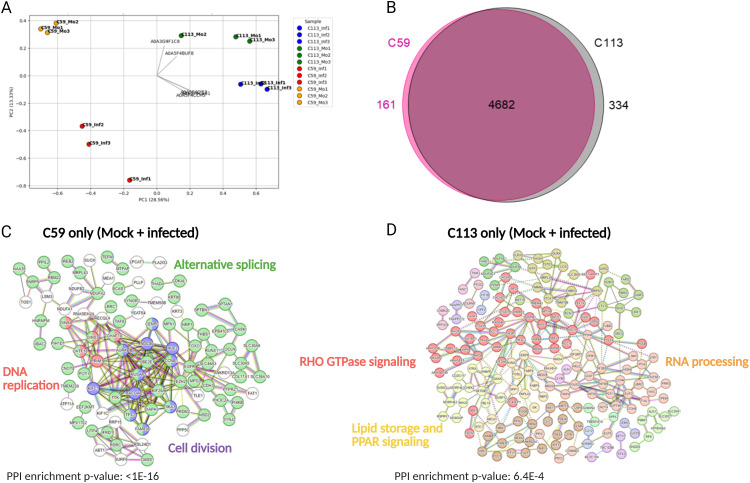
Comparison of protein expression of the MDCK cell clones C59 and C113. A: principal coordinate analysis of all detected proteins across the three replicates of C59 infected (C59_Inf), C59 mock (C59_Mo), C113 infected (C113_Inf) and C113 mock (C113_Mo) using Bray-Curtis distance (S1 File). B: Venn diagram of all detected proteins (C59 and C113) in at least two of the three replicates per condition. C, D: protein network analysis of proteins, which are only expressed by one cell clone using the STRING platform and *H. sapiens* as the reference proteome. Each protein is assigned to the corresponding gene. Colors refer to functional clusters, which are connected with straight line, genes of different clusters with a functional similarity are connected with a dashed line. Genes that belong to two functional clusters are marked with two different colors. The name of the corresponding functional cluster pathways is written in the same color as the genes are marked. The PPI enrichment p-value is a statistical measure of the probability that a connection occurs randomly.

As an initial step a principal coordinate analysis (PCoA) was performed (S1 File). As illustrated in Fig 4A, the three replicates of all four sample sets are closely grouped, indicating that the two cell lines and the two cell states (mock versus infected) exhibit distinct patterns of protein expression. Interestingly, for C113, protein expression of the three infected replicates seems to be more similar to each other compared to the infected replicates of C59 (Fig 4A).

Across all samples, 5507 proteins were identified, 5177 of which could be detected in at least two replicates of the C59 or C113 clones. Of the 5177 proteins, about 6.5% (334) were exclusively detected in C113, 90% (4682) in both cell lines and 3.5% (161) in C59 only (Fig 4B) (S2 File).

The proteins, which were detected in only one of the two cell clones, were subsequently analyzed using the protein network platform STRING. *H. sapiens* was selected as the host due to the more comprehensive information available about the proteins. However, we ensured that each gene name was annotated to the same protein for both *C. l. familiaris* and *H. sapiens*. As shown in S2 Fig, for *H. sapiens,* more information regarding cross-connections between proteins is available, resulting in a more interconnected network. Moreover, for all comparisons of Fig 4, protein-protein interaction enriched p-values were below 0.05, indicating that the connections between the proteins were not random (string-db.org). For proteins detected exclusively in the C59 clone, the majority were associated with alternative splicing (Fig 4C). In contrast, proteins detected only in the C113 clone, could be functionally assigned to Ras homologue (RHO) GTPase signaling and lipid storage (Fig 4D). Alternative splicing was been demonstrated to play a pivotal role in the proliferation of cancer cells. This process enhances the efficiency of the energy metabolism and can prevent apoptosis [37]. In addition, RHO GTPase signaling is a key regulator of actin and microtubule dynamics and can influence numerous processes in cancer cells such as membrane trafficking, cell cycle and phosphoinosit 3-Kinase (PI3K) signaling. Furthermore, it can facilitate viral processes during infection. It is especially important for virus entry due to its involvement in clathrin-mediated endocytosis and its effects on viral gene expression [38,39].

**Fig 5:**
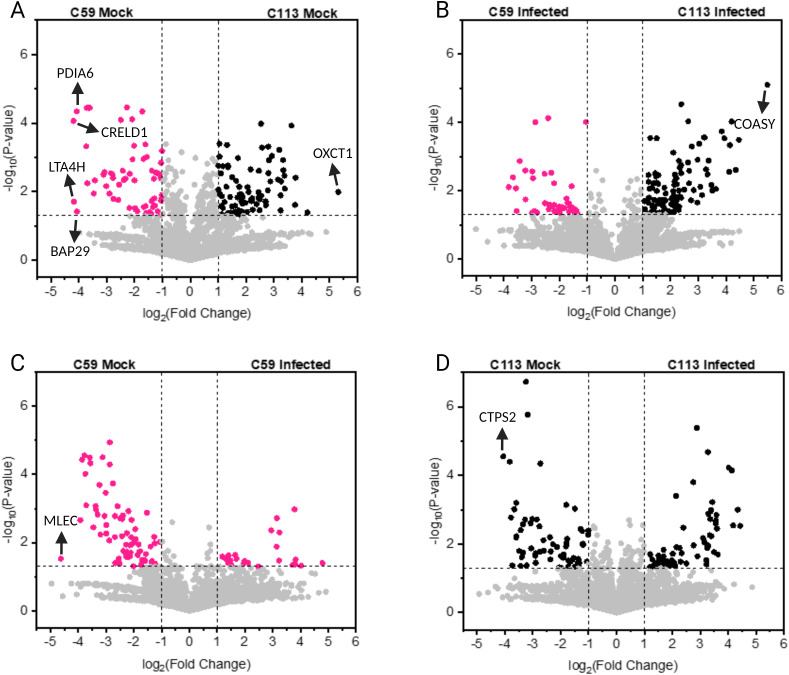
Comparison of the protein expression profiles of the MDCK cell clones C59 and C113 – without infection (mock, Mo) and after infection (Inf). A-D: volcano plots; for the calculation of the fold-change, average ratios of mock (Mo) or infected (Inf) were calculated for all proteins detected in all replicates, respectively. Each point corresponds to one protein. Proteins outside the lines were significantly different expressed (p-value <0.05) with a fold change of >2 (grey lines). Statistical significance was calculated with student’s t test (two-sided, heterogeneous variances).

In the next step, we compared differences in the amount of the expressed proteins the sample sets (Fig 5A–D), i.e., samples without infection (mock) and after infection. For the mock samples, 266 of the 5177 detected proteins in both cell lines were significantly different expressed (119 upregulated in C59, 147 upregulated in C113). With a 40-fold increase in expression in the C113 clone, the succinyl-CoA:3-ketoacid-coenzyme A transferase (Acc: A0A8I3MS17, Gene: OXCT1) was the most upregulated protein compared to C59. This protein is a key enzyme for ketone metabolism, sterol production, tumorigenesis and signaling in tissues and cancer cells [40,41]. In relation to that finding, higher levels of sterols were found in the C113 clone compared to C59. In the case of the C59 mock infection, four of the proteins that were upregulated were in a similar range with an about 16–18 fold higher abundancy than in the C113 clone. Two of these are classified as protein-disulfide isomerases which have been shown to have a positive effect on the proliferation of cancer cells (Acc: A0A8I3Q0F7, Gene: PDIA6, Bai et al., 2019; Acc: A0A8I3MZE2, Gene; CRELD1, [42]), one as an aminopeptidase with antiviral effects (Acc: A0A8I3PTW1, Gene: LTA4H, [43]) and one as a B-cell receptor-associated protein, for which the function has not yet been described (Acc: A0A8I3PYE6, Gene: BAP29, [44]).

For the infected samples, 179 proteins were found to be significantly upregulated in the C59 (57) or in the C113 (122) cell clone (Fig 5B). Again, for the C113 clone, the bifunctional coenzyme A synthase, exhibited a 45-fold increase in expression compared to C59. The bifunctional coenzyme A synthase (Acc: A0A8I3N4F7, Gene: COASY) is involved in the cofactor biosynthesis for multiple lipids [45]. Interestingly, this protein has been described as a positive regulator of PI3K signaling [46], a process that has been shown to promote IAV infection during both early and late stages [47].

A comparative analysis of the changes after infection of each cell line individually (Fig 5C, D) revealed that for the C113 clone three times more proteins were significantly upregulated compared to C59 (100 vs. 31). In contrast, the number of downregulated proteins showed more similarity (94 vs. 76). For the C113 clone, multiple proteins were either up- or downregulated during infection. For C59, two proteins were identified with very high changes in expression levels: the cytidine triphosphate (CTP) synthase 2 (Acc: A0A8I3QAX6, Gene: CTPS2) was downregulated 25-fold, whereas the rRNA methyltransferase 1 (Acc: A0A8I3Q638, Gene: MRM1) was upregulated 28-fold. CTPS2 is associated with cell proliferation [48], whereas MRM1 plays a crucial role in RNA modification [49].

Subsequently, a protein network analysis was performed on the significantly differentially expressed proteins (student’s t-test, p < 0.05; log2 fold changes >1 and <−1) to identify patterns and cross connections. To this end, all significantly differently expressed proteins from each comparison (C113Mo vs. C59Mo, C113Inf vs. C59Inf) were analyzed using the functional protein expression network tool STRING (reference proteome: *H. sapiens*). The protein networks and related pathways are shown in the Supplementary (S2 Fig).

**Fig 6:**
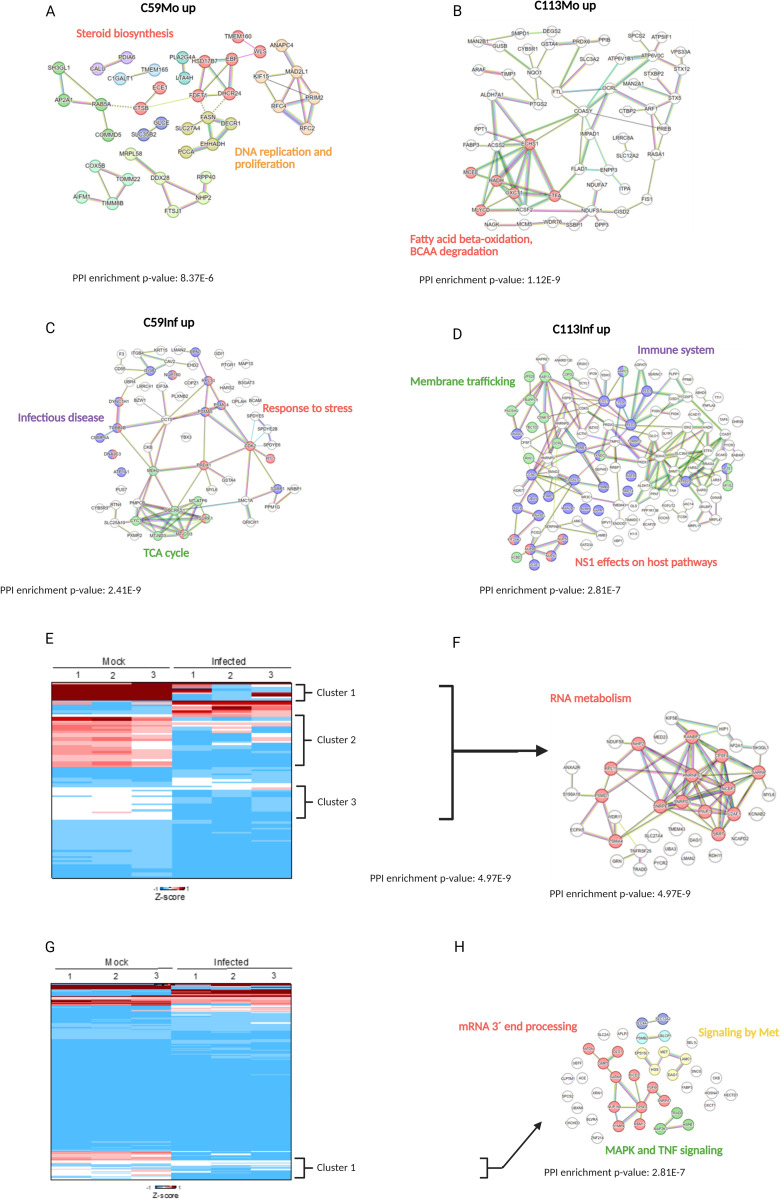
Analysis of significantly differentially expressed proteins of the C59 and C113 MDCK cell clones. Statistically significant changes (Students t-test; p-values<0.05, log2 fold change < −1 or >1) between the two monoclonal cell lines C59 and C113 are shown. A-D: reactome pathways enriched for the comparison of C59 vs. C113 mock (uninfected) and C59 vs. C113 infected. Proteins separated into up- and downregulated in each cell line - C59Mo up and C113 Mo up= upregulated in C59 Mock or C113 Mock; C59Inf up and C113 Inf up= upregulated in C59 infected or C113 infected. Protein network analysis was performed using the STRING platform and *H. sapiens* as the reference proteome. Each protein is assigned to the corresponding gene. Colors refer to functional clusters, which are connected by straight lines, genes of different clusters with a functional similarity are connected by dashed lines. Genes that belong to two functional clusters are marked with two different colors. The name of the corresponding pathway of functional clusters is labeled in the same color as the genes. The PPI enrichment p-value is a statistical measure of the probability that a connection occurs randomly. E, G: heat maps for C59 and C113 were gnerated with the three replicates based on significant differences (Students t-test, p < 0.05) for the z-scores using OriginLab software. Protein network analysis of the clusters for proteins downregulated in C59 infected (F) and in C113 infected **(H)**.

A comparison of the protein networks of the two cell clones without infection (mock), reveals differences in fatty acid metabolism and DNA replication (Fig 6A,B, S2 Fig). While the steroid biosynthesis was found to be more active in the C59 clone compared to C113, the fatty acid beta oxidation and the degradation of specific amino acids was upregulated in C113. Among the various steroids, cholesterol has been identified as a significant player. Cholesterol biosynthesis has been demonstrated to be beneficial for the proliferation of cancer cells and the replication of IAV, where it plays a pivotal role for cell survival and viral budding [50–53]. Other steroids are also described to act as antivirals inside the cell [54]. The second group of proteins, which was found to be upregulated in the uninfected C59 cells (mock) is related to DNA replication and proliferation. The majority of these proteins have been found to be associated with cell cycle progression and mitosis. Therefore, the faster growth of the C59 clone may be ascribed to this observation given that these proteins promote cell proliferation [55].

In addition, an upregulation of fatty acid beta-oxidation and a degradation of branched chain amino acids (BCAAs) such as valine, leucine and isoleucine was observed for the C113 clone (mock, Fig 6B). Both changes in the metabolism are described for mice, which are highly suspectable to IAV infection [56]. With regard to cell proliferation, an increase in beta oxidation can excert a positive or negative influence on cancer cells, as described previously [57]. Conversely, BCAA degradation has been shown to be associated with reduced growth through a reduced glucose uptake [58].

The data of the infected cells demonstrate that certain proteins related to infectious disease, stress response and tricarboxylic acid (TCA) cycle were upregulated in the C59 compared to the C113 clone (Fig 6C). A notable finding was the observation that the majority of proteins associated with stress response and infectious disease exhibited a significant overlap, suggesting a substantial interference between these two pathways. Given the broad nature of these two terms, a search was conducted for 15 genes related to infectious disease and stress response using the Reactome pathway database. This investigation revealed that three genes (NUP160, RPL30, DNAJC3) are directly linked to IAV RNA transcription and replication. Three other genes (PSMA5, PSMC4, RPN1) were linked to Wingless-related integration site (WNT) signaling, proliferation and DNA replication and another three genes (TUBB4B, CHMP4A, DYNC1H1) to vesicle-mediated transport. However, the association of the gene products with multiple signaling pathways complicates the evaluation of their impact (PSMA5 [59], PSMC4 [60], RPN1 [61]). For example, in one study it was shown, that activation of WNT signaling was positively correlated with IAV replication [62]. Liu et al. discussed that WNT signaling was downregulated during IAV infection [60]. Nevertheless, the observed hits for proliferation and DNA replication could be indicative of the tendency of the C59 clone for ongoing growth even during IAV PR8 infection.

In contrast, proteins of the C113 clone exhibited an increase in the expression of proteins related to membrane trafficking and the cell-mediated immunity. Four of the proteins associated with the latter were found to be directly linked to IAV NS1 (Fig 6D). In addition, previous studies demonstrated that the three nuclear pore complex proteins (NUP43, 58, 98) are involved in the transport between nucleus and cytoplasm by different viruses [63–65]. Apart from that, also antiviral pathways were triggered (Fig 6D). Specifically, RNAse L (RNASEL) and the RNA-activated kinase (EIF2AK2) have been identified as key players in antiviral responses, known to activate interferon pathways [66–68]. However, the impact of interferon signaling on IAV replication in MDCK cells is very low [69].

Membrane trafficking plays a critical role in the late stages of IAV infection, particularly during virus assembly and budding. During this step, the Ras-related protein Rab-11A (Rab11A), which has the highest number of cross-connections to other proteins in this study (Fig 5D), seems to be a key factor for the synthesis of new virus particles [70,71].

To facilitate a comparative analysis of the infected and the uninfected state (mock), heat maps were generated based on significant differences (t-test, p < 0.05) in average z-values across the three replicates (Fig 6E,G). For the C59 clone, three clusters were identified, exhibiting a decrease in protein expression following infection. Based on all proteins from these clusters, a network can be derived that is mainly related to RNA metabolism (Fig 5F). It was evident that these proteins function as integral components of diverse processes, including anaplastic lymphoma kinase (ALK) signaling, splicing, and virus replication (S2 Fig). For the C113 clone, apart from RNA processing, MET, mitogen-activated protein kinase signaling (MAPK) and tumor necrosis factor (TNF) were also downregulated after infection (Fig 6G), all involved in antiviral effects and inhibition of IAV replication [72–74].

**Fig 7:**
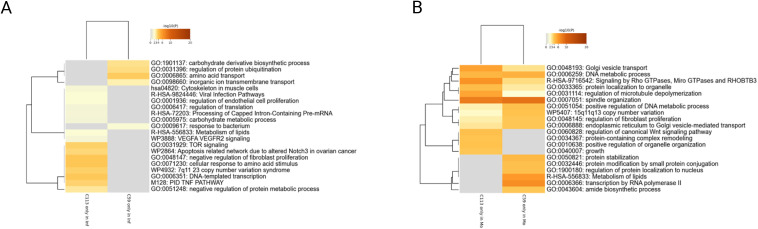
Metascape analysis of proteins detected in the C59 or C113 MDCK cell clones. A) proteins detected only in infected C59 and C113 (C59 only in Inf, C113 only in Inf), or B) proteins detected only in mock C59 and C113 (C59 only in Mo, C113 only in Mo) samples (n = 3). Corresponding genes were mapped on the human genome and enriched as described in Zhou et al., 2019 [28] using the default settings of the web tool.

Next, we analyzed proteins that were exclusively expressed in infected cell clones or in mock samples (Fig 7A,B). For the C59 clone, only 26 proteins were present in all three infected replicates, whereas for C113, a total of 88 “unique” proteins were identified. These proteins were enriched and aligned to the corresponding pathways using the web-based portal Metascape [28]. For the C59 clone, the proteins were linked to carbohydrate metabolism, protein ubiquitination and the transport of amino acids and ions. In contrast, for C113, they were linked to different signaling processes such as apoptosis, TNF, VEGF and TOR signaling, as well as cytoskeleton and viral infection pathways. Notably, the lipid and carbohydrate metabolism were also affected by IAV repplication. Regarding the latter, an increase in the activity of the central carbohydrate and lipid metabolism is described to be benefical for efficient IAV replication since key proteins and structural parts of the virus are produced in higher quantities [75]. Especially during the late phase of IAV infection, TOR signaling and initiation of apoptosis by IAV are described to be beneficial for efficient virus assembly and release [76,77]. These discrepancies with the C59 clone may provide evidence to support the hypotheses that a key distinction between the two monoclonal cell lines lies is the efficient assembly and release of IAV PR8 particles.

A large number of proteins (379) were not identified in the infected samples of C59 compared to C113 (198 for C113 compared to C59). Many of these proteins are associated with related pathways, including Golgi transport, DNA metabolism and microtuble depolymerisation (Fig 7B). For C59, lipid metabolism and RNA polymerase II transcription appear to be inactivated following IAV infection, while for C113 proteins related to cell growth and regulation of WNT signaling were only present in the mock samples. In addition to the pivotal role of lipids in the replication of IAV, it is known that the cellular RNA polymerase II interacts with the RNA polymerase of IAV forming a complex for efficient replication of the virus [78]. The observed downregulation of this process may consequently impact the replication of the IAV genome, potentially resulting in reduced titers for the C59 clone. For C113, the shutdown of WNT signaling could limit IAV PR8 titer, as it is described that this signaling pathway can have pro-viral effects, especially in early stages of infection [63].

In addition to analyzing the signaling pathways, we examined host proteins related to glycosylation to determine whether differences in the glycosylation of IAV PR8 HA and NA could explain variations in virus titer and infectivity. Therefore, the proteins involved in the *N*-glycosylation were extracted from the data and marked on the corresponding KEGG pathway map (S2–S3 Figs). Eleven proteins of the *N*-glycosylation pathway were identified in both cell clones. Of these proteins, chitobiosyldiphosphodolichol beta-mannosyltransferase (ALG1) was higher expressed after infection in C113, while beta-1,4-galactosyltransferase 1 (B4GALT1) was expressed at significantly lower levels in C113. No significant changes in the expression of proteins related to *N*-glycosylation were observed in C59. ALG1 is involved in the initial steps of glycosylation in the cytoplasm, while B4GALT1 is located in the Golgi apparatus and involved in the synthesis of more complex glycosylation structures [79,80]. ALG1 has also been described as being involved in the glycosylation of IAV HA protein [81]. Therefore, overexpressing ALG1 could be beneficial for producing infectious IAV PR8 particles, while downregulating B4GALT1 after IAV PR8 infection could result in HA and NA proteins with simpler glycan structures. This has been shown to improve the infectivity of viral particles by McCullers [82].

**Fig 8:**
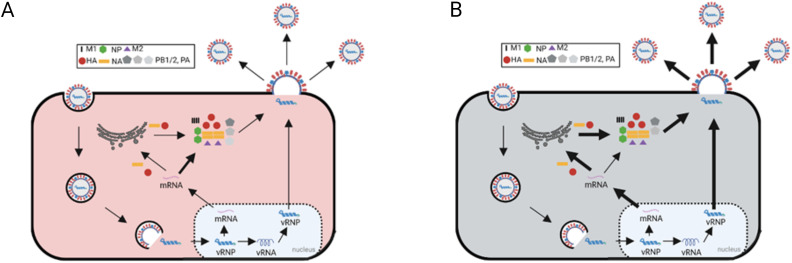
Summary of changes in the C59 (A) and C113 (B) cell clones following IAV infection. Thicker arrows show a increase of this process by host-cell proteins.

A comprehensive summary of all changes after infection of the two monoclonal cell lines is given in Fig 8. For the C113 clone, a number of antiviral intracellular signaling pathways were found to be downregulated during infection. In contrast, for C59, the JAK/STAT and the RAS pathways were upregulated. This could result in a limitation of the production and release of IAV PR8 particles in this cell line compared to C113. However, given that both cell lines produced significant amounts of IAV particles, it is evident that several virus-favouring factors were also activated. For the C59 clone, there is a high probability of increased viral compound production due to the highly active ribosomal activity, TCA and cholesterol synthesis pathways. Conversely, the C113 clone exhibited an increased presence of cellular factors that facilitate the efficient export of vRNPs and vmRNAs as well as posttranslational modifications. It is conceivable that the observed differences in IAV PR8 titers may be attributable to an insufficent virus budding and release in the C59 clone in comparison to C113.

Overall, a comprehensive analysis was conducted to identify key characteristics between a fast-growing and a high-producing clonal MDCK cell line. The results obtained facilitated the identification of potential bottlenecks of IAV PR8 replication and engendered a more profound comprehension of effective IAV production in animal cell cultures. Increased membrane trafficking, activation of TOR signaling and apoptosis, and a reduction in antiviral signaling appear to be pivotal in fostering the high-yield production of IAV in MDCK cells.

## Supporting information

S1 Fig DIA-PASEF scan windows. Windows were selected based on DDA measurements using py diAID (Version 0.030) as described in Skowronek et al. [25] resulting in a precursor coverage of 99.80%. Mobility (1/K0) and m/z areas were adjusted in a manner that enabled the capture of the majority the ions, particularly those with a high density.
(DOCX)

S2 Fig Protein network analysis of C59 infected and mock. Protein networks were generated using the platform STRING for the comparison of C59 and C113 Mock (C59 Mo, C113 Mo) and infected (C59 Inf, C113 Inf). A, B: comparison of the two databases C. l. familiaris and H. sapiens. Each protein is assigned to the corresponding gene. The PPI enrichment p-value is a statistical measure of the probability that a connection occurs randomly. C, D, E, F: enriched reactome pathways for C59 and C113 mock-infected and infected. G: enriched reactome pathways for the three clusters of downregulated proteins during infection in C59 (referred to Fig 5E).
(DOCX)

S3 Fig KEGG pathway mapping of N-glycan biosynthesis of C59 and C113. Respective proteins found for C59 (red) and C113 (blue) at 12 hpi were shown for the KEGG pathway by using the KEGG color tool. Significant up- or downregulation is indicated by an arrow below the respective protein and color for the cell line.
(DOCX)

S1 Table Target peptides for HA, NA, NP, M1 and NS1 protein quantification. Underlined amino acids were C^13^ labelled and N^15^ labelled (adapted from [22]).
(DOCX)

S2 Table Errors of the absolute quantification of HA, NP, NA, M1 and NS1 proteins. Technical error: percentage standard deviation of the abundance of all peptides divided through the absolute protein copy number (n = 3); biological error: percentage standard deviation of three replicates.
(DOCX)

S3 Table Limit of detection (LoD) and limit of quantification (LoQ) for the HA and NP protein. (DOCX)

S1 File Python code for principal component analysis. (DOCX)

S2 File Proteins and their abundancies, those visualized in volcano plots and heatmaps. (XLSX)
