# Supplementary material for: Quantitative analysis of proteomic changes in two monoclonal suspension MDCK cell lines infected with human influenza A virus (H1N1)
Source: PLoS One. 2025 Oct 21;20(10):e0327939. doi: 10.1371/journal.pone.0327939 (PMC12539711; doi:10.1371/journal.pone.0327939)
Supplement: S1 Fig — Windows were selected based on DDA measurements using py diAID (Version 0.030) as described in Skowronek et al. [25] resulting in a precursor coverage of 99.80%. Mobility (1/K0) and m/z areas were adjusted in a manner that enabled the capture of the majority the ions, particularly those with a high density. (DOCX) [file pone.0327939.s001.docx]

| **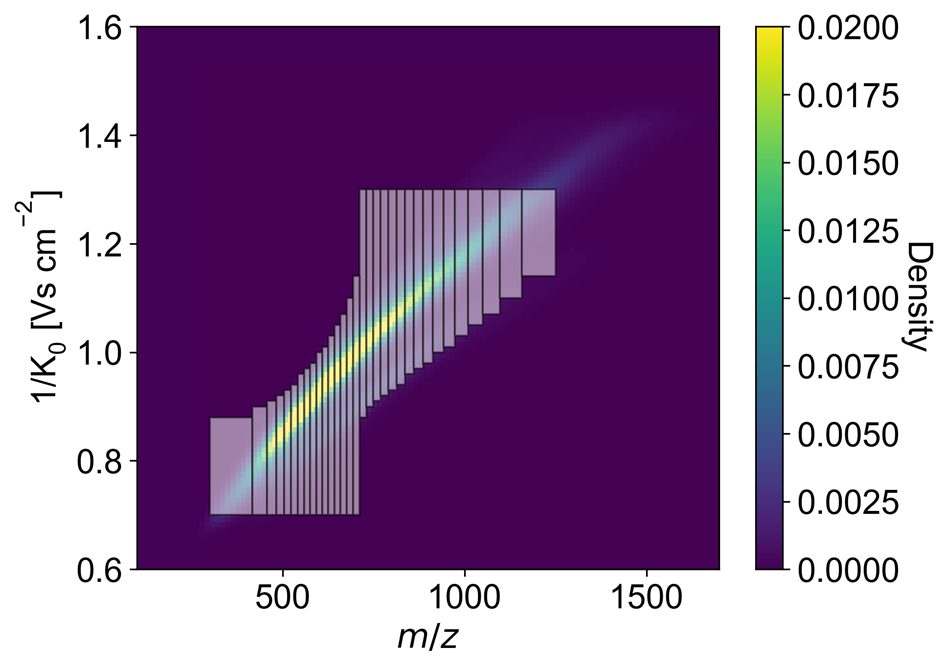** |
| --- |

**Figure S1: DIA-PASEF scan windows.** Windows were selected based on DDA measurements using py diAID (Version 0.030) as described in Skowronek et al. [25] resulting in a precursor coverage of 99.80 %. Mobility (1/K_0_) and m/z areas were adjusted in a manner that enabled the capture of the majority the ions, particularly those with a high density.
