# Supplementary material for: Quantitative analysis of proteomic changes in two monoclonal suspension MDCK cell lines infected with human influenza A virus (H1N1)
Source: PLoS One. 2025 Oct 21;20(10):e0327939. doi: 10.1371/journal.pone.0327939 (PMC12539711; doi:10.1371/journal.pone.0327939)
Supplement: S2 Fig — Protein networks were generated using the platform STRING for the comparison of C59 and C113 Mock (C59 Mo, C113 Mo) and infected (C59 Inf, C113 Inf). A, B: comparison of the two databases C. l. familiaris and H. sapiens. Each protein is assigned to the corresponding gene. The PPI enrichment p-value is a statistical measure of the probability that a connection occurs randomly. C, D, E, F: enriched reactome pathways for C59 and C113 mock-infected and infected. G: enriched reactome pathways for the three clusters of downregulated proteins during infection in C59 (referred to Fig 5E). (DOCX) [file pone.0327939.s002.docx]

**A**

| **B**  **C59Mo_up *H. sapiens***  **C59Mo_up *C. l. familiaris*** |
| --- |
| 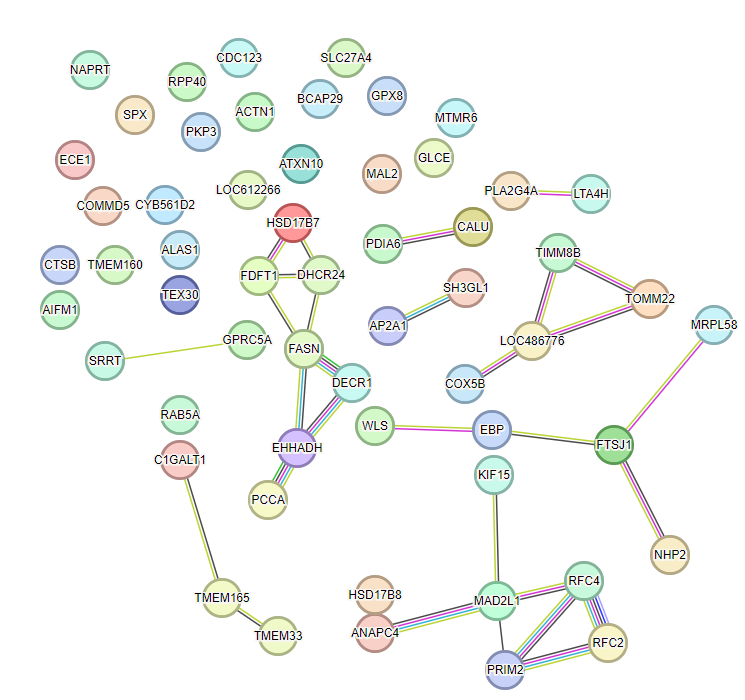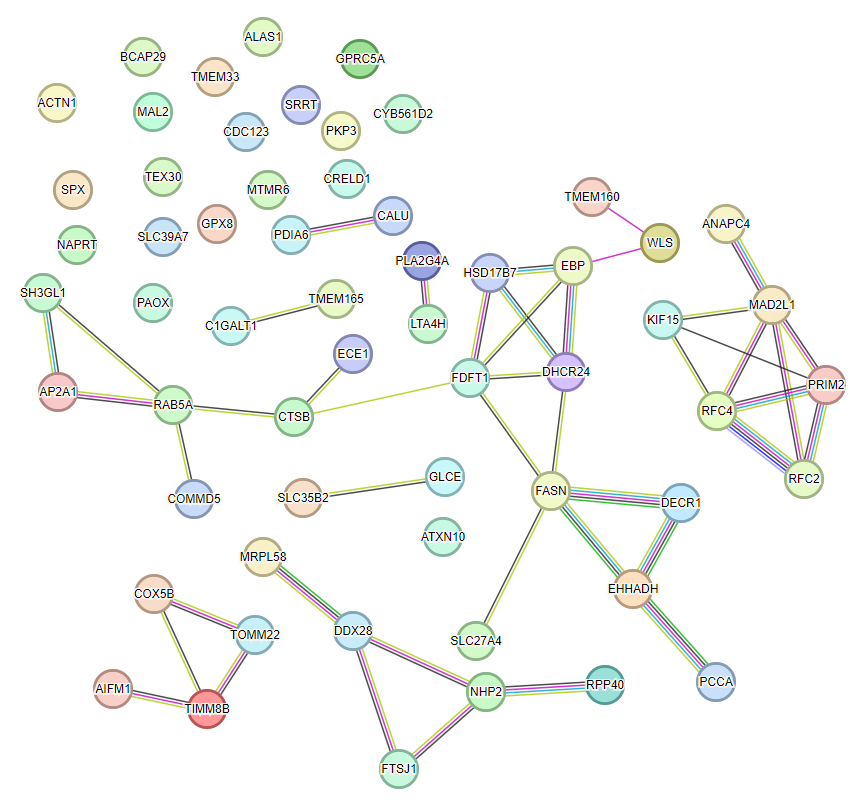  PPI enriched p-value: 8.09E-4  **D**  **C**  **C113_C59Mo – C113 up**  PPI enriched p-value: 8.36E-6  **C59_C113Mo_C59up** |
|  |
| 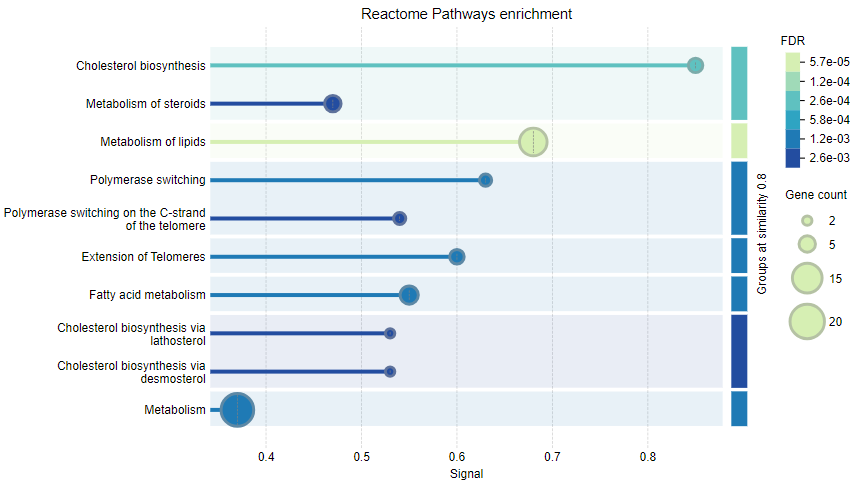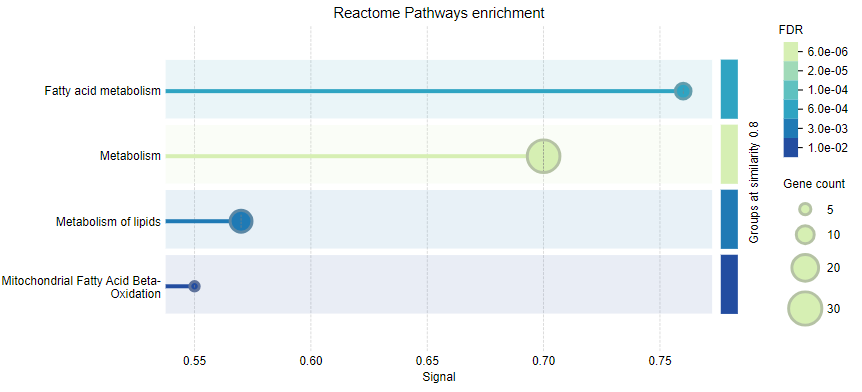  **F**  **E** |
|  |
| 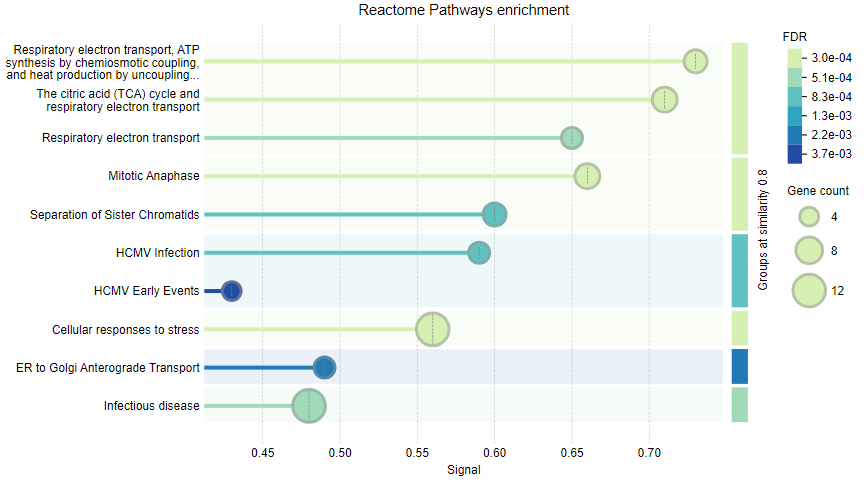 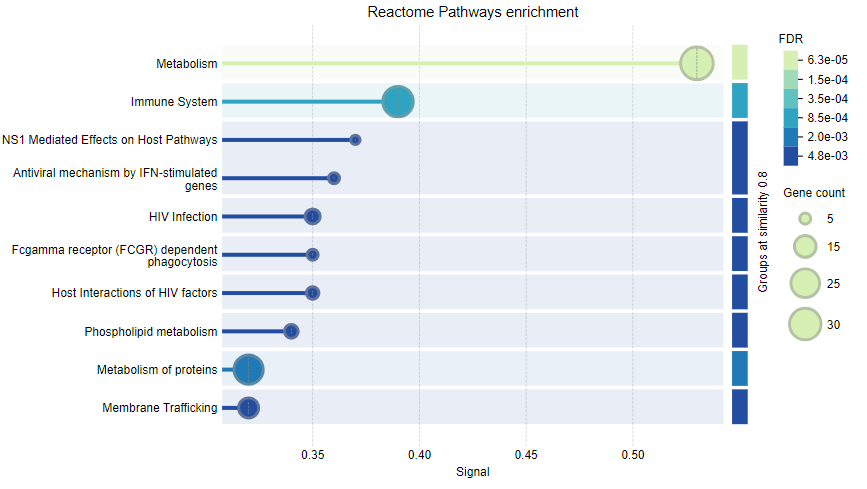  **C59_C113Inf_C113up**  **C59_C113Inf_C59up** |
|  |
| 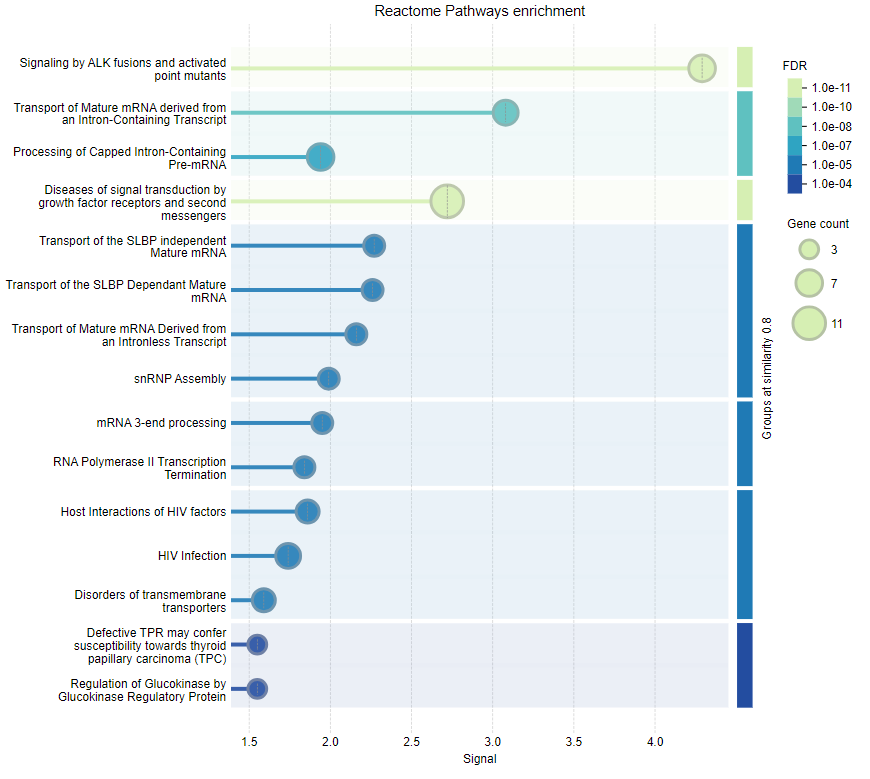 |

**Figure S2: Protein network analysis of C59 infected and mock.** Protein networks were generated using the platform STRING for the comparison of C59 and C113 Mock (C59 Mo, C113 Mo) and infected (C59 Inf, C113 Inf). A, B: comparison of the two databases *C. l. familiaris* and *H. sapiens* . Each protein is assigned to the corresponding gene. The PPI enrichment p-value is a statistical measure of the probability that a connection occurs randomly. C, D, E, F: enriched reactome pathways for C59 and C113 mock-infected and infected. G: enriched reactome pathways for the three clusters of downregulated proteins during infection in C59 (referred to fig 5E).

**G**
