# Supplementary material for: Quantitative analysis of proteomic changes in two monoclonal suspension MDCK cell lines infected with human influenza A virus (H1N1)
Source: PLoS One. 2025 Oct 21;20(10):e0327939. doi: 10.1371/journal.pone.0327939 (PMC12539711; doi:10.1371/journal.pone.0327939)
Supplement: S3 Fig — Respective proteins found for C59 (red) and C113 (blue) at 12 hpi were shown for the KEGG pathway by using the KEGG color tool. Significant up- or downregulation is indicated by an arrow below the respective protein and color for the cell line. (DOCX) [file pone.0327939.s003.docx]

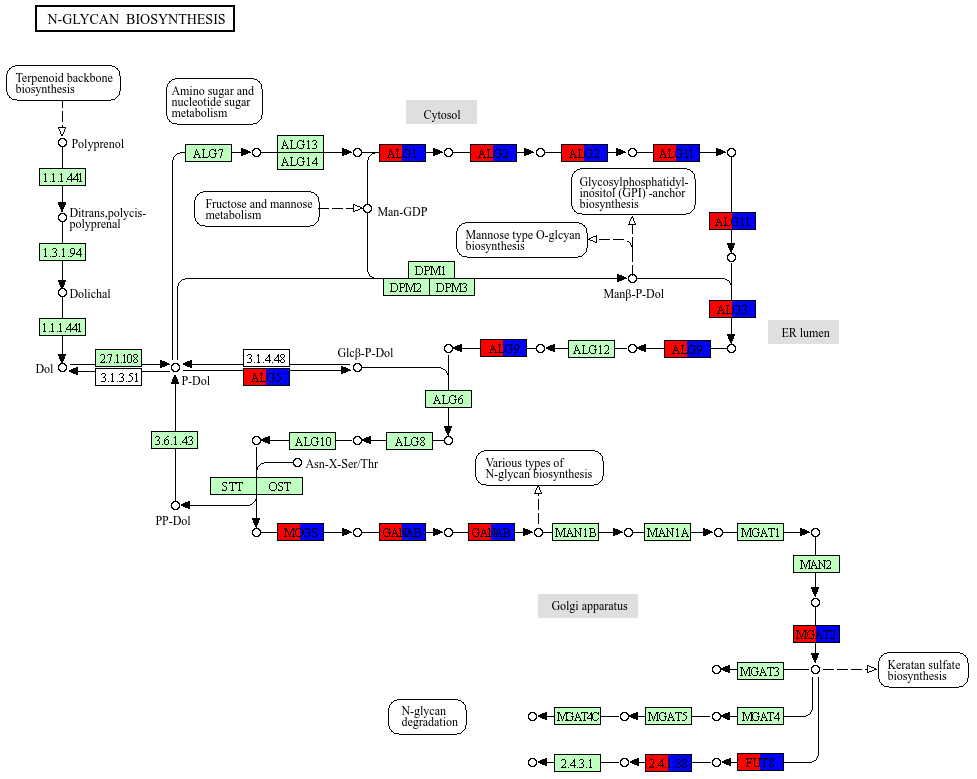


**Figure S3:** **KEGG pathway mapping of N-glycan biosynthesis of C59 and C113.** Respective proteins found for C59 (red) and C113 (blue) at 12 hpi were shown for the KEGG pathway by using the KEGG color tool. Significant up- or downregulation is indicated by an arrow below the respective protein and color for the cell line.

**G**
