# Supplementary material for: Quantitative analysis of proteomic changes in two monoclonal suspension MDCK cell lines infected with human influenza A virus (H1N1)
Source: PLoS One. 2025 Oct 21;20(10):e0327939. doi: 10.1371/journal.pone.0327939 (PMC12539711; doi:10.1371/journal.pone.0327939)
Supplement: S1 Table — Underlined amino acids were C13 labelled and N15 labelled (adapted from [22]). (DOCX) [file pone.0327939.s004.docx]

**Table S1:** **Target peptides for HA, NA, NP, M1 and NS1 protein quantification.** Underlined amino acids were C^13^ labelled and N^15^ labelled (adapted from [22]).

| **Protein** | **Abbreviation** | **Peptide sequence** | | | **m/z** | **AQUA m/z** |
| --- | --- | --- | --- | --- | --- | --- |
| Hemagglutinin (HA) | EIG | EIGNGCFEFYH**K** | | | 750.8328 | 754.8399 |
|  | STQ | STQNAINGITN**K** | | | 630.8314 | 634.8385 |
|  | TLD |  | TLDFHDSNV**K** | 588.2881 592.2959 | | |
|  | EQL | EQLSSVSSFE**R** | | | 634.8095 | 639.8136 |
|  | FTP | FTPEIAERP**K** | | | 594.3246 | 598.3317 |
| Neuraminidase (NA) | DGT | DGTGSCGPVYVDGANGV**K** | | | 876.8969 | 880.9040 |
|  | YNG | YNGIITETI**K** | | | 576.3196 | 580.3267 |
|  | YGN | YGNGVWIG**R** | | | 511.2649 | 516.2691 |
|  | EPF | EPFISCSHLEC**R** | | | 767.8425 | 772.8466 |
|  | ALM | ALMSCPVGEAPSPYNS**R** | | | 918.4244 | 923.4285 |
| Nucleoprotein (NP) | EGY | EGYSLVGIDPF**R** | | | 676.8465 | 681.8506 |
|  | LIQ | LIQNSLTIE**R** | | | 593.8437 | 598.8479 |
|  | GVF | GVFELSDE**K** | | | 512.2539 | 516.2610 |
| Matrix Protein 1 (M1) | LED | LEDVFAG**K** | | | 439.7351 | 443.7422 |
|  | TRP | TRPILSPLT**K** | | | 563.3537 | 567.3608 |
|  | QMV | QMVTTTNPLI**R** | | | 637.3507 | 642.3544 |
|  | TIG | TIGTHPSSSAGL**K** | | | 628.3357 | 632.3428 |
| Non-structural protein 1 (NS1) | VAD | VADQELGDAPFLD**R** | | | 773.3808 | 778.3850 |
|  | GST | GSTLGLDIETAT**R** | | | 667.3515 | 672.3557 |
|  | NAV | NAVGVLIGGLEWNDNTV**R** | | | 964.0052 | 969.0094 |
