# Supplementary material for: Quantitative analysis of proteomic changes in two monoclonal suspension MDCK cell lines infected with human influenza A virus (H1N1)
Source: PLoS One. 2025 Oct 21;20(10):e0327939. doi: 10.1371/journal.pone.0327939 (PMC12539711; doi:10.1371/journal.pone.0327939)
Supplement: S2 Table — Technical error: percentage standard deviation of the abundance of all peptides divided through the absolute protein copy number (n = 3); biological error: percentage standard deviation of three replicates. (DOCX) [file pone.0327939.s005.docx]

**Table S2:** **Errors of the absolute quantification of HA, NP, NA, M1 and NS1 proteins.** Technical error: percentage standard deviation of the abundance of all peptides divided through the absolute protein copy number (n=3); biological error: percentage standard deviation of three replicates.

|  | **Technical error** | **Biological error** |
| --- | --- | --- |
| **Hemagglutinin (HA)** | 30 % | 16 % |
| **Nucleoprotein (NP)** | 38 % | 21 % |
| **Neuraminidase (NA)** | 37 % | 26 % |
| **Matrix protein 1 (M1)** | 34 % | 22 % |
| **Non-structural protein 1 (NS1)** | 20 % | 14 % |
| **Average** | 32 % | 20 % |
