# Supplementary material for: Quantitative analysis of proteomic changes in two monoclonal suspension MDCK cell lines infected with human influenza A virus (H1N1)
Source: PLoS One. 2025 Oct 21;20(10):e0327939. doi: 10.1371/journal.pone.0327939 (PMC12539711; doi:10.1371/journal.pone.0327939)
Supplement: S3 Table — (DOCX) [file pone.0327939.s006.docx]

**Table S3: Limit of detection (LoD) and limit of quantification (LoQ) for the HA and NP protein.**

|  | **LoD [copies/cell]** | **LoQ [copies/cell]** |
| --- | --- | --- |
| **Hemagglutinin (HA)** | 5.8E+03 | 2.3E+04 |
| **Nucleoprotein (NP)** | 1.4E+04 | 5.6E+04 |
