# Supplementary material for: Quantitative analysis of proteomic changes in two monoclonal suspension MDCK cell lines infected with human influenza A virus (H1N1)
Source: PLoS One. 2025 Oct 21;20(10):e0327939. doi: 10.1371/journal.pone.0327939 (PMC12539711; doi:10.1371/journal.pone.0327939)
Supplement: S1 File — (DOCX) [file pone.0327939.s007.docx]

Python code for principal component analysis

import pandas as pd

import numpy as np

from sklearn.preprocessing import StandardScaler, RobustScaler, MaxAbsScaler

from scipy.spatial.distance import pdist, squareform

from sklearn.decomposition import PCA

import matplotlib.pyplot as plt

import seaborn as sns

# Schritt 1: CSV-Datei laden

# Dateipfad anpassen

file_path = 'Gene_PCA_neu.csv'

data = pd.read_csv(file_path, index_col=0, sep=";")

#data = data.dropna(how='all', inplace=True)

data

# Farben explizit für jeden Sample-Namen festlegen

sample_colors = {

    'C113_Inf1': 'blue', 'C113_Inf2': 'blue', 'C113_Inf3': 'blue',

    'C113_Mo1': 'green','C113_Mo2': 'green','C113_Mo3': 'green',

    'C59_Inf1': 'red','C59_Inf2': 'red','C59_Inf3': 'red',

    'C59_Mo1': 'orange','C59_Mo2': 'orange','C59_Mo3': 'orange',

    # Weitere Sample-Namen und zugehörige Farben hier hinzufügen

}

scalers = [StandardScaler(), RobustScaler(), StandardScaler(), MaxAbsScaler()]

metrics = ["braycurtis"]

### be careful with execution (e.g., for qunatile comparision)

for scaler in scalers:

    for dist_metric in metrics:

        # Schritt 2: Daten skalieren (optional, je nach Daten)

        #scaler = StandardScaler() #RobustScaler / StandardScaler /MaxAbsScaler

        data_scaled = scaler.fit_transform(data.T) # Transponieren, um Samples in den Zeilen zu haben

        #data_scaled = data.T # kein scaling

        print(type(data_scaled))

        # Schritt 3: Distanzmatrix berechnen (z. B. euklidische Distanz, Bray-Curtis wäre auch möglich)

        #dist_metric="cosine"

        dist_matrix = squareform(pdist(data_scaled, metric=dist_metric)) # if scaling aplied

        #dist_matrix = squareform(pdist(data.T, metric='euclidean'))

        # Schritt 4: PCoA durchführen

        # PCoA ist eine PCA auf der Distanzmatrix

        pcoa = PCA(n_components=2)

        pcoa_result = pcoa.fit_transform(dist_matrix)

        # Schritt 6: Loadings der Features berechnen und die wichtigsten auswählen

        feature_loadings = pcoa.components_.T * np.sqrt(pcoa.explained_variance_)

        num_top_features = 5  # Anzahl der anzuzeigenden Features

        top_features_idx = np.argsort(np.abs(feature_loadings[:, 0]) + np.abs(feature_loadings[:, 1]))[-num_top_features:]

        top_features = data.index[top_features_idx]  # Namen der Top-Features

        top_loadings = feature_loadings[top_features_idx]

        # Schritt 5: Ergebnis visualisieren

        # Ergebnisse in DataFrame für Plot

        pcoa_df = pd.DataFrame(pcoa_result, columns=['PC1', 'PC2'])

        pcoa_df['Sample'] = data.columns  # Sample-Namen hinzufügen

        # Farben dem DataFrame hinzufügen

        #pcoa_df['Color'] = pcoa_df['Sample'].map(sample_colors)

        # Plot erstellen mit den spezifischen Farben

        plt.figure(figsize=(10, 7))

        sns.scatterplot(

            x='PC1', y='PC2',

            data=pcoa_df,

            s=100,

            hue='Sample',

            palette=sample_colors,  # Definierte Farben verwenden

            edgecolor='k',

        )

        title = 'Principal Coordinates Analysis (PCoA) ('+str(scaler) +"," + str(dist_metric) +" distance)"

        plt.title(title)

        plt.xlabel(f'PC1 ({pcoa.explained_variance_ratio_[0]*100:.2f}%)')

        plt.ylabel(f'PC2 ({pcoa.explained_variance_ratio_[1]*100:.2f}%)')

        plt.legend(title='Sample', bbox_to_anchor=(1.05, 1), loc='upper left')

        # Schritt 8: Pfeile der wichtigsten Features hinzufügen

        for i, feature in enumerate(top_features):

            plt.arrow(0, 0,top_loadings[i, 0], top_loadings[i, 1],  width=0.00001, color='gray', alpha=0.7, head_width=0.0002)

            plt.text(top_loadings[i, 0]*1.15, top_loadings[i, 1]*1.15, feature, color='black', ha='center', va='center')

        for i in range(len(pcoa_df)):

            plt.text(

                pcoa_df['PC1'][i],

                pcoa_df['PC2'][i],

                pcoa_df['Sample'][i],

                horizontalalignment='left',

                size='medium',

                color='black',

                weight='semibold'

            )

        plt.grid(True)

        fname= "PCoA_"+str(scaler)+ "("+dist_metric+").png"

        plt.savefig(fname, dpi='figure', format="jpeg")

        plt.show()
